# Parental personality, mental health, and fear of happiness as predictors of perceived coparenting relationship quality among mothers and fathers of preschoolers

**DOI:** 10.1080/00049530.2023.2205537

**Published:** 2023-05-03

**Authors:** Cynthia A. Frosch, Marcus A. Fagan, Wendy Middlemiss, Joohee G. Kim, Sheila R. Sjolseth

**Affiliations:** a Department of Human Development and Family Science, Auburn University, Auburn, USA; b Center for Research Design and Analysis, Texas Woman’s University, Denton, USA; c Department of Educational Psychology, University of North Texas, Denton, USA

**Keywords:** Anxiety, coparenting, depression, fear of happiness, parents, personality

## Abstract

**Objective:**

Existing theoretical models and research findings highlight individual parent characteristics as contributors to coparenting relationship quality. Yet less is known about how indices of parental personality, beliefs, and mental health symptoms relate to coparenting perceptions among parents of preschoolers. This study examines direct and indirect paths connecting parents’ Big Five personality traits, fear of happiness, and depression and anxiety symptoms with perceived coparenting quality.

**Method:**

Using an online survey design, 160 parents (81 mothers; 79 fathers) of preschoolers (age 2–5 years) completed the Ten-Item Inventory of Personality, Fear of Happiness Scale, Patient Health Questionnaire-4, and Coparenting Relationship Scale – Brief Form.

**Results:**

Parental Emotional Stability negatively related to anxiety and depressive symptoms and to fear of happiness. Anxiety symptoms and fear of happiness directly, and negatively, related to coparenting quality. Emotional stability was indirectly, positively related to coparenting quality via lower levels of parental anxiety and fear of happiness. Although parental depressive symptoms were unrelated to coparenting quality, parents’ Openness to Experience and fear of happiness positively predicted depressive symptoms.

**Conclusion:**

Findings suggest parents’ anxiety symptoms and fear of happiness may underlie the processes by which parental Emotional Stability relates to perceived coparenting quality among parents of preschoolers.

## Key Points

**What is already known about this topic:**

1. Coparenting relationships are important for children’s social, emotional, and cognitive development.
2. Coparenting relationships are separate and distinct from the marital or intimate partner relationships.
3. Existing theory and research highlights parent characteristics including personality, beliefs, and emotions as contributors to coparenting quality.

**What this topic adds:**

1. Parents higher on Emotional Stability reported fewer anxiety and depression symptoms, less fear of happiness, and higher coparenting quality.
2. The path between Emotional Stability and coparenting quality was explained by parental fear of happiness.
3. Emotional Stability was also indirectly related to coparenting quality, via parental anxiety, but not depression symptoms.

Extensive research documents the importance of coparenting relationship quality for children’s attachment relationships (Brown et al., 2010), behavioural (e.g., Feinberg et al., 2014) and social development (e.g., Zhao et al., 2020), as well as academic skills and school readiness (Cabrera et al., 2012). The coparenting relationship represents a family subsystem that provides unique information for understanding child outcomes, beyond individual parent contributions to child development (e.g., Karreman et al., 2008). While existing theoretical and conceptual models of coparenting highlight parental characteristics as contributors to relationship quality (e.g., Feinberg, 2003), few studies have examined multiple maternal and paternal characteristics within a single study, and among parents of preschoolers. This study examines the pathways linking parental personality, fear of happiness, and depression and anxiety symptoms to perceived coparenting quality among parents of 2 to 5-year-old children, a time in family life when coparenting may be important for understanding children’s executive functioning along with the development of later behaviour problems (see Parkes et al., 2019).

## Background

Coparenting reflects the efforts of at least two adults who work together to care for a child (e.g., McHale & Lindahl, 2011). In this way, coparenting is tied directly to the parental role and sharing of parental responsibilities (Feinberg, 2003). In early childhood, coparenting relationships are particularly important for understanding parenting behaviour, as well as children’s socio-emotional development and competence (e.g., Feinberg, 2002). Moreover, consideration of coparenting during the early childhood years is critical for understanding externalizing behaviour problems later in childhood (e.g., Parkes et al., 2019).

Coparenting relationships are separate and distinct from marital relationships (e.g., Feinberg, 2003) and coparenting relationships do not require a marital partnership. In fact, shifting demographic profiles including an increase in unmarried, cohabitating parents raising children and coparenting while apart given increases in divorce (Pew Research Center, 2018), and high rates of relationship dissolution in the first years of a child’s life (see Goldberg & Carlson, 2015, for discussion) necessitate consideration of diverse coparenting experiences.

The concept of coparenting aligns with family systems theory, which posits the family is composed of interdependent parts and subsystems (Cox & Paley, 1997, 2003; Minuchin, 1985). Coparenting relationships represents an executive subsystem within a family (e.g., Dollberg et al., 2021; Reader et al., 2017; Zvara et al., 2019), and the functioning of this subsystem is “characterized by healthy interparental functioning (i.e., positive coparenting quality) as well as healthy functioning of the individuals who comprise it” (Reader et al., 2017, p. 453). Research and theory suggest multiple indices of parental functioning, including parents’ personalities, beliefs, and affect, may contribute to the quality of coparenting relationships (e.g., Feinberg, 2003; Schoppe-Sullivan & Mangelsdorf, 2013).

### Parental personality

Although theories of personality differ, and approaches to the measurement of personality traits are diverse, the Five Factor Model of personality has been cited widely and used in studies of individual differences. Focusing on five broad dimensions (i.e., Big Five) – Neuroticism, Extraversion, Conscientiousness, Agreeableness, and Openness to Experience (see McCrae & John, 1992; Tupes & Christal, 1961) – the Five Factor Model predicts a range of individual and relational outcomes. As summarized by John (2021), Agreeableness describes compassion, altruism, trust and respect, while Conscientiousness considers personal responsibility, productivity, and organization. Extraversion reflects social energy, including sociability, approach, and assertiveness while Neuroticism describes negative emotionality, including depression, anxiety, and anger. Finally, Openness reflects originality, open-mindedness, and “complexity of an individual’s mental and experiential life” (p. 60).

Despite evidence that parental personality, typically conceptualized as Big Five personality traits (e.g., Effenberger et al., 2021; Oliver et al., 2009), (a) impacts family relationships (Stright & Bales, 2004), (b) predicts indices of parental well-being, including parental burnout (Le Vigouroux et al., 2017), and (c) relates to a range of parenting perceptions and behaviours (Prinzie et al., 2009), surprisingly little empirical research has focused on maternal and paternal personality and coparenting quality. Existing evidence has been minimal and mixed. For example, Schoppe-Sullivan and Mangelsdorf (2013) found paternal negative emotionality predicted later undermining coparenting behaviour. Similarly, Laxman et al. (2013) found direct and interactive effects of parental negative emotionality and observed coparenting behaviour, although the association was negative for mothers and positive for fathers.

Moreover, although Stright and Bales (2004) found maternal personality was related to observed unsupportive coparenting behaviour (e.g., competing, criticizing, contradicting) during family interaction, understanding if/how each personality trait contributes to coparenting was not the focus of their study; instead, an overall personality adjustment score was created based on parents’ report on the five subscales of the Revised NEO Personality Inventory (Costa & McCrae, 1992). What remains unclear is how Big 5 personality characteristics relate to coparenting quality and if this link is explained by parental beliefs and emotions (i.e., fear of happiness; anxiety and depression symptoms).

### Fear of happiness

Research on adult well-being has long recognized the impact of personality on satisfaction with life (e.g., Hayes & Joseph, 2003), happiness, and affect (e.g., DeNeve & Cooper, 1998). One aspect of happiness, fear of happiness, encompasses a relatively stable set of beliefs (Belen et al., 2020; Joshanloo, 2013b) about the experience, expression, and desirability of happiness (Joshanloo, 2018), including the belief different types of happiness should be avoided because happiness can lead to bad things happening (Joshanloo & Weijers, 2014; Joshanloo, 2013a). Fear of happiness may negatively impact mental health (Blasco-Belled et al., 2021) and relate to dampening or decreased experiences of positive affect and mood (Yildirim & Belen, 2018). For example, fear of happiness predicts greater stress, anxiety, and depression (Gilbert et al., 2014; see also Jordan et al., 2020). Moreover, fear of happiness relates to adult attachment styles, with insecure attachment associated with higher fear of happiness (Joshanloo, 2018). This is interesting from a theoretical perspective, given that attachment security provides a sense of safety in the world (i.e., secure base; see Vaughn, 2018), and a foundation for (a) the development of positive internal working models of self and others across the lifespan (see Crowell, 2021) and (b) the perception and experience of emotion, including positive emotions (see Gilbert et al., 2014). Fear of happiness also appears to influence reported life satisfaction (Joshanloo, 2013b), suggesting the potential value of exploring this construct within the family system.

Accordingly, we posited fear of happiness would relate to Big 5 personality traits, mental health symptoms, and to relationship satisfaction, defined here as perceived coparenting relationship quality. Drawing from the extant literature, we suggest fear of happiness may be an important, understudied variable that relates to individual well-being and coparenting quality. For example, parents’ ability to recognize, accept, and enhance positive emotions (socialization of positive affect) may impact child well-being (see Katz et al., 2014), and fear of experiencing positive emotions may have important clinical implications (Gilbert et al., 2014). However, fear of happiness has largely been examined as an individual construct within university student populations, or adults, regardless of parenting status (e.g., Belen et al., 2020; Joshanloo & Weijers, 2014; Joshanloo, 2013b, 2018). Examining fear of happiness among parents may help with identification of mothers and fathers at-risk for decreased individual and relational well-being across the experience of parenthood.

### Mental health symptoms

Studies investigating parental characteristics and coparenting quality also underscore the value of considering parental mental health. For example, Williams (2018) found maternal and paternal depression related to less optimal coparenting, with persistent effects over time. Tissot et al. (2016) found mothers’ reports of higher levels of depressive symptoms were associated with lower levels of observed coparenting support during mother-father-infant play. In their recent study with Swedish fathers, Lee et al. (2021) found fathers’ reports of greater coparenting quality on the Brief Coparenting Relationship Scale (Feinberg et al., 2012) were associated with lower depressive symptoms.

While more extensive attention has been devoted to depression in comparison to anxiety, Majdandžić et al. (2012) called for expanded recognition of how parental anxiety contributes to, or amplifies, coparenting quality. Indeed, a recent study of Israeli mothers and fathers with preschool-age children found parental anxiety was negatively associated with perceptions of coparental support, agreement, and closeness, and positively associated with perceived undermining and competition (Dollberg et al., 2021). Taken together, these studies suggest the need to further examine the link between parental mental health and coparenting quality.

## Present study

This investigation seeks to broaden understanding of the determinants of coparenting relationship quality and inform intervention efforts by focusing on parental mental health and well-being (e.g., Feinberg & Kan, 2008) during a time of enduring stress and change (e.g., Feinberg et al., 2021), such as parenting young children. Two primary aims guided the study: (a) exploration of direct relations between Big Five personality traits, anxiety and depressive symptoms, fear of happiness, and perceived coparenting quality among parents of preschool-age children, and (b) examination of possible indirect pathways via fear of happiness and parental mental health symptoms in the associations between parental personality and perceived coparenting quality.

Drawing from the extant literature, we expected parents lower on Emotional Stability (i.e., higher Neuroticism) to report lower coparenting quality. Moreover, parents reporting higher depressive symptoms and higher anxiety symptoms were expected to report lower coparenting quality. We further hypothesized parents higher on fear of happiness would report lower coparenting quality. Conceptually, we suggest parents with different personalities would perceive the coparenting relationship differently via differences in their fears of happiness and mental health symptoms. Parents higher in Neuroticism may experience greater fear of happiness, as adults higher in Neuroticism experience greater anxiety, impulsivity, and hostility, worry and fear (John & Srivastava, 1999; McCrae & Costa, 1992), and consequently, report lower coparenting quality. Thus, parental fear of happiness and mental health symptoms (anxiety and depression) were expected to be indirect mechanisms linking parental Emotional Stability (low Neuroticism) and perceived coparenting quality.

## Method

### Participants

This research received ethical approval from the University of North Texas (IRB 17–373). Inclusion criteria were being a parent of a child aged 2–5 years, English speaking, and residing within the United States. Participants were recruited as individuals, rather than as coparenting dyads, thus each parent in the study was from a different family. Of the 205 participants recruited for a larger study of parenting, 172 (83.9%) reported currently sharing parenting responsibilities (i.e., coparenting) with another individual. Thus, 33 participants were removed from the sample because coparenting was not applicable to their family. Twelve additional participants were excluded from analyses because they (a) did not complete the survey, (b) answered uniformly on a measure, or (c) failed the two attention checks in the survey.

Among the participants in the analytic sample (*n* = 160; 79 fathers) 22% identified as a racial or ethnic minority individual within the United States (5.0% Hispanic, 4.4% Black, 4.4% Asian; and 8.2% mixed ethnicity/race). The remaining 78% identified as White. Participants reported that they were married (80%), single, living as married (11.9%), single, never been married (6.9%), or separated (1.2%). Table 1 presents demographic information. No significant differences were found between the original coparenting sample (*N* = 172) and the analytic sample (*n* = 160).

**Table 1.** Descriptive statistics (*N* = 160).

	Mothers (*n* = 81)	Fathers (*n* = 79)
Variable	Mean (*SD*)	Mean (*SD*)
Coparenting	66.69 (16.23)	72.65 (10.48)
Fear of Happiness	2.90 (4.78)	3.93 (5.10)
Openness to Experience	10.03 (2.79)	10.70 (2.44)
Extraversion	7.48 (4.02)	8.15 (4.10)
Agreeableness	11.73 (2.37)	10.98 (2.93)
Conscientiousness	12.26 (2.13)	12.24 (2.12)
Emotional Stability	9.85 (3.30)	12.19 (2.42)
Depression	0.86 (1.48)	0.60 (1.40)
Anxiety	1.52 (1.60)	0.74 (1.25)
Age	36.78 (6.87)	36.71 (7.03)
Child Sex	60.5% Female (49)	36.7% Female (29)
Income		
< $25,000	3.7% (3)	1.3% (1)
$25k – $40k	19.8% (16)	15.2% (12)
$40k – $60k	19.8% (16)	15.2% (12)
$60k – $80k	27.2% (22)	24.1% (19)
$80k – $100k	17.3% (14)	8.9% (7)
$100,000+	12.3% (10)	35.4% (28)

*SD* = Standard Deviation.

### Procedure

Participants were recruited via Amazon’s Mechanical Turk (MTurk), an established crowdsourcing platform for data collection (e.g., Buhrmester et al., 2011; Lovett et al., 2018; Mason & Suri, 2012; Peer et al., 2014) that can be both efficient (Berinsky et al., 2012; Mason & Suri, 2012) and valid (Lovett et al., 2018). Strategies used to support data quality included: (1) requesting experienced, high-reputation MTurk workers/respondents (i.e., workers with an approval rating above 95%; e.g., Peer et al., 2014), (2) offering fair compensation for survey completion, (3) aligning estimated time for survey completion with workers’ actual time spent on survey completion (e.g., Lovett et al., 2018), and (4) incorporating attention checks into the survey (Hauser & Schwarz, 2016). After consenting online to participate, participants completed a Qualtrics survey focused on parent, preschooler, and family characteristics. Following survey completion, participants were compensated $5.50, a rate equivalent to approximately 40 minutes of work at the United States federal minimum hourly wage of $7.25.

### Measures

#### Ten Item Personality Inventory (TIPI)

The TIPI (Gosling et al., 2003) is a 10-item, self-report measure of the five factors of personality: *Emotional Stability* (Reverse coded *Neuroticism*), *Extraversion, Openness to Experience, Agreeableness, and Conscientiousness*. Each factor was measured by two items using a 7-point Likert scale (1 = *strongly disagree*; 7 = *strongly agree*). Sample items include “I see myself as extraverted, enthusiastic” and “I see myself as open to new experiences, complex”. The TIPI has been translated into multiple languages and demonstrates adequate test-retest reliability (see Nunes et al., 2018) and convergent validity to the International Personality Item Pool, Five Factor Model (Ehrhart et al., 2009).

#### Patient health questionnaire for depression and anxiety (PHQ-4)

Depression and anxiety symptoms were assessed using the PHQ-4, a reliable, valid, and brief screener for clinical settings and general population samples (e.g., Kroenke et al., 2009; Löwe et al., 2010). Participants were asked how frequently they had been “bothered by the following problems” over the past 2 weeks. Items included “feeling down, depressed or hopeless” (depression; 2 items), and “feeling nervous, anxious or on edge” (anxiety; 2 items). Participants responded using a 4-point scale (0 = *not at all*; 3 = *nearly every day*).

#### Fear of happiness scale

This 5-item measure assesses fear of happiness (i.e., happiness aversion) with each item rated on a 7-point scale (1 = *strongly disagree*; 7 = *strongly agree*; see Joshanloo, 2013b; measure used with permission). Sample items include “I prefer not to be too joyful, because usually joy is followed by sadness” and “Excessive joy has some bad consequences”. The factor structure and partial scalar invariance of the Fear of Happiness Scale has been validated across cultures (Joshanloo et al., 2014). The coefficient alphas for the current sample were .86 (mothers) and .81 (fathers).

#### Coparenting relationship scale – brief form

The 14-item brief form of the Coparenting Relationship Scale (Feinberg et al., 2012) was used. Participants described the ways they worked with their coparent to parent the child using a scale ranging from 0 (*not true of us*) to 6 (*very true of us*). Sample items include “I believe my partner is a good parent” and “My partner and I have the same goals for our child”. Parents also reported on how frequently, within the company of the other coparent, they engaged in behaviours such as “Argue with your partner about your child, in the child’s presence” using a scale ranging from 0 (*never*) to 6 (*several times a day*). A total coparenting quality score was created by summing the items scores, taking into account required reverse scoring. The validity and reliability of the CRS has been demonstrated; the brief form provides a strong representation of the larger subscale scores (Feinberg et al., 2012). For this sample, coefficient alphas were .92 (mothers) and .86 (fathers).

### Plan of analysis

All variables were examined for normality, homogeneity of variance, presence of outliers, and missing values (see Table 1 for descriptive statistics). To examine interrelatedness of study variables, bivariate correlations were calculated using the Statistical Package for Social Sciences (SPSS). Next, using Mplus, path analysis was conducted in predicting coparenting quality by fear of happiness, anxiety, depression, and Big Five personality traits. Then, fear of happiness, anxiety, and depression were explored as indirect variables linking personality to coparenting quality. Finally, each path was tested for parental role differences (mother; father) in relation to coparenting quality. Inquiries about study data should be directed to the first or second authors.

## Results

Bivariate correlations are presented in Table 2. As hypothesized, parents lower on Emotional Stability and higher on fear of happiness and anxiety symptoms reported lower coparenting quality. Moreover, parental Agreeableness and Conscientiousness correlated positively with reported coparenting quality. Contrary to hypotheses, parents’ depression symptoms were unrelated to reported coparenting quality.

**Table 2.** Bivariate correlations among study variables.

	1	2	3	4	5	6	7	8	9
Coparenting Quality	-								
Fear of Happiness	−.32**	-							
Openness to Experience	.11	−.06	-						
Extraversion	.07	−.19*	.38**	-					
Agreeableness	.22**	.22**	.08	.07	-				
Conscientiousness	.18*	−.11	.19*	.16*	.22**	-			
Emotional Stability	.27**	−.21**	.32**	.43**	.27**	.44**	-		
Depression	−.14	.38**	−.30**	−.30**	−.00	−.09	−.34**	-	
Anxiety	−.25**	.25**	−.37**	−.37**	−.10	−.13	−.55**	.60**	-

*p* < .05*; *p* < .01**.

In the path analysis, the overall direct model was just-identified (no degrees of freedom), given that the number of estimated paths and the number of elements in the covariance matrix are the same, *p* < .001; CFI = 1.00, RMSEA <.001, SRMR <.001, with two statistically significant pathways explaining 28.1% variance in coparenting quality. These pathways partially supported hypotheses, with fear of happiness, b = −0.78, *t*(159) = −3.539, *p* < .001, and anxiety, b = −0.77, *t*(159) = −4.95, *p* < .001, as two significant, negative predictors of coparenting quality.

**Figure 1.**
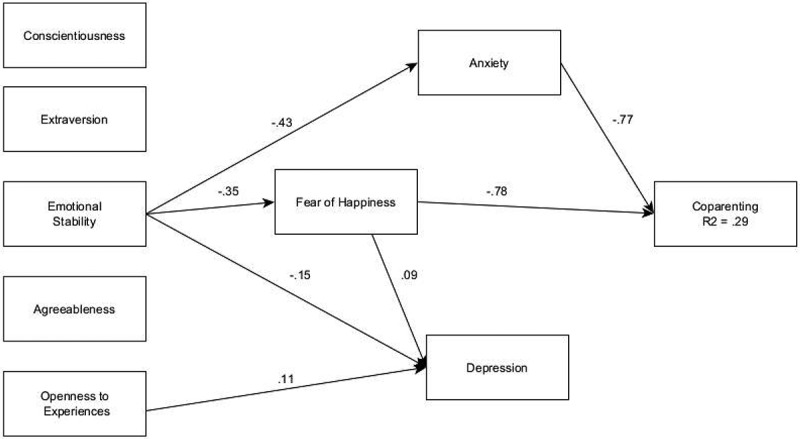
Path analysis final model of personality, fear of happiness, anxiety and depression to coparenting.

Next, fear of happiness and anxiety and depression symptoms were explored as mediators of Big Five personality traits with anxiety and depression symptoms also mediating the relationship between fear of happiness and coparenting quality (Figure 1). Results showed one significant direct effect to fear of happiness from Emotional Stability, b = −.35, *t*(159) = −2.850, *p* = .004, which resulted in one significant indirect path from Emotional Stability through fear of happiness, b = 0.27, *t*(159) = 2.22, *p* = .026. Additionally, Emotional Stability had two significant, negative direct paths to depression symptoms, b = −0.15, *t*(159) = −4.466, *p* < .001, and anxiety symptoms, b = −.43, *t*(159) = −2.538, *p* = .011, that resulted in one significant indirect path through anxiety symptoms to coparenting quality, b = .33, *t*(159) = 2.259, *p* = .024. Moreover, Openness to Experience, b = .11, *t*(159) = 2.819, *p* = .005, and fear of happiness, b = .09, *t*(159) = 4.519, *p* < .001, both had a positive relationship to depression symptoms. Combined, this resulted in explaining 28.6% of the variance in coparenting quality, χ^2^(12) = 18.888, *p* = .091; CFI =.935, RMSEA =.060, SRMR =.052 (see Figure 1). Next, parameters were sequentially freed to potentially identify parent role differences (mothers; fathers); however, no differences were identified.

## Discussion

By looking across multiple parental characteristics (personality, fear of happiness, anxiety and depression symptoms) within one study, the current work extends the literature via three major contributions. First, this research begins to examine fear of happiness as a variable related to parents’ reports of personality, mental health symptoms, and coparenting quality. We found fear of happiness had both a direct and indirect role in perceived coparenting quality. Second, our finding of direct and indirect pathways between parental personality and multiple indices of well-being and coparenting quality offers a new contribution to the literature. Specifically, the relationship between Emotional Stability and coparenting quality was indirect via parental anxiety symptoms and fear of happiness. Third, by engaging a sample of mothers and fathers of preschool children within the United States, this investigation moves the study of fear of happiness beyond assessment of college students and adults, without consideration of parental role. Here, the paths linking parental personality, mental health, and fear of happiness to perceived coparenting quality did not differ for mothers and fathers.

### Parental personality

Findings extend the limited and mixed literature on the connection between parental personality and coparenting quality (Laxman et al., 2013). Results from our path analysis suggest Emotional Stability may be particularly relevant for understanding perceived coparenting quality among parents of preschoolers. Specifically, mothers and fathers who were higher on Emotional Stability (i.e., low on Neuroticism) reported higher coparenting quality. Given that central issues for parents of young children include discipline and supporting children’s social competence (Tompkins & Villaruel, 2022), autonomy, and self-control (e.g., Meuwissen & Carlson, 2015), perhaps higher Emotional Stability extends to a parent’s greater ability to manage emotions within the family system, thereby contributing to perceptions of higher coparenting quality.

Although we did not find direct paths between other personality traits and coparenting, future research may benefit from considering similarities or differences in partners’ personality characteristics. For example, Schoppe-Sullivan et al. (2018) found parents with similarly-low levels of Extraversion exhibited less coparenting competition. Moreover, additional exploration of the connection between parental Openness to Experience and depression symptoms is needed. Perhaps for parents reporting higher Openness, the tasks and demands of parenting and coparenting young children limit their ability to pursue new opportunities and areas of intellectual curiosity and interest, thereby relating to higher depression symptoms, perhaps in interaction with child temperament. As Koenig et al. (2010) note, examination of parental Openness warrants additional research; consideration of facets of Openness in relation to depressive symptoms (Khoo & Simms, 2018) may also be useful.

### Fear of happiness and mental health symptoms

Results from our study indicated a strong, inverse association between fear of happiness and coparenting perceptions. Perhaps higher fear of happiness creates greater hesitancy in parenting decisions or more frequent engagement in gatekeeping behaviour (defined as efforts to control and encourage/discourage the coparent’s relationship with the child; Schoppe-Sullivan & Altenburger, 2019). This, in turn, may contribute to perceptions of poorer coparenting quality.

Parental anxiety was also directly and strongly related to perceived coparenting quality. This extends Majdandžić et al. (2012), who summarized the limited existing literature and noted that, for fathers and possibly mothers, higher levels of anxiety were associated with lower levels of coparenting support. One possible interpretation for our findings is that higher levels of anxiety and fear of happiness may make a parent guarded in sharing parenting tasks and labour with their partner, which contributes to lower quality coparenting. Alternatively, when one coparent does not share decision making or support the other coparent, that may increase the coparent’s experience of anxiety and fear. Future longitudinal research could uncover such bidirectional processes.

Although parental depression symptoms were unrelated to coparenting perceptions, depression symptoms were directly related to fear of happiness. Finding a significant link between depression symptoms and fear of happiness is consistent with recent work by Blasco-Belled et al. (2021), who found that, among college students, greater depression was associated with greater fear of happiness. Uniquely, however, the present study extends the literature on the link between depression and fear of happiness to a sample of mothers and fathers with preschool age children.

Finally, we found partial support for our hypothesis that parents with different personalities would perceive the coparenting relationship differently, via differences in their fears of happiness and mental health symptoms. As expected, parental fear of happiness was a variable indirectly linking parental Emotional Stability and coparenting quality. In addition, there was an indirect path linking Emotional Stability and coparenting quality via parental anxiety (not depression) symptoms. Thus, fear of happiness and anxiety symptoms may be particularly important for understanding how Emotional Stability relates to coparenting perceptions among parents of preschoolers. As Negrini (2020) notes, the integration of supports for coparenting into service delivery, intervention, and clinical protocols during infancy and early childhood may improve child outcomes and the functioning of family systems across generations. Our ability to understand how parents’ individual characteristics contribute to their coparenting perceptions may provide a valuable starting place to build awareness and self-compassion as a foundation for individual and relational well-being (Lathren et al., 2021).

### Strengths and limitations

Strengths of this investigation included inclusion of mothers and fathers of preschool-age children and a focus on previously underexplored constructs that may advance understanding of coparenting. Another strength was the study’s recruitment strategy, which allowed for parents with a variety of coparenting arrangements to participate. We viewed this approach as critical for capturing the range of coparenting relationships parenting partners may share, as well as diversity family structures. However, we did not recruit multiple coparenting partners within a family and our sample size was too limited to examine whether differences in coparenting arrangements impacted how parental personality, mental health symptoms, and fear of happiness relate to coparenting perceptions; these will be important next steps for research. For example, Lamela and Figueiredo (2016) aptly noted that “marital dissolution does not dissolve the family” (p. 334); thus research examining coparenting experiences during and following relationship formation, dissolution, and critical family transitions is needed. Moreover, because our sample was limited to parents within the United States, examination of direct and indirect paths between parental characteristics and coparenting quality would benefit from a wider cultural lens. Finally, although we discuss direct and indirect paths from a statistical perspective, we collected data at only one time point; thus, longitudinal examination is clearly necessary.

## Conclusion

Moving forward, examining the interrelations among multiple indices of parent functioning may be informative in supporting parents and coparents during a demanding time of parenting, such as the early childhood years. Particularly, this examination could be meaningfully considered through the lens of network psychometrics, which recognizes mutualistic interactions between each parent, including behavioural and environmental factors (Marsman & Rhemtulla, 2022; van der Maas et al., 2006). As these components together create a dynamic system, an analysis via network psychometrics would be valuable from a research and practice perspective. As Blasco-Belled et al. (2021) note, targeting negative beliefs about happiness via community-based and therapeutic interventions may be beneficial. We further suggest the field of early childhood mental health, which situates the child in an environment of relationships and recognizes the critical importance of building caregiver capacity (National Scientific Council on the Developing Child, 2008/2012), may benefit from conversations with coparents about their fears of happiness and how those fears may relate to their mental health, personality, and family functioning. Given that major life events (i.e., the birth of a child) may have an everlasting effect on personality traits (Bleidorn et al., 2018), this presents a window of opportunity for these conversations to improve coparenting quality through improvement of emotional stability and lessening of fear of happiness.

Given that adult attachment style has been identified as a factor related to fear of happiness (e.g., Gilbert et al., 2014; Joshanloo, 2018), future research exploring adult attachment representations may elucidate the processes by which parental personality relates to fear of happiness and contributes to psychosocial and relational functioning (i.e., coparenting) among parents of young children. Moreover, future studies may benefit from inclusion of child temperament, as the associations between parental personality and coparenting quality may be moderated by child temperament (e.g., Laxman et al., 2013). Despite the limitations of the present study, our findings suggest future studies of coparenting may benefit from inclusion of parental fear of happiness and examination of pathways between multiple parental characteristics and coparenting quality for mothers and fathers.
